# Early transient dysautonomia predicts the risk of infantile epileptic spasm syndrome onset: A prospective cohort study

**DOI:** 10.3389/fneur.2022.1090155

**Published:** 2022-12-22

**Authors:** Ipsita Goswami, Bryan Maguire, Vann Chau, Emily W. Tam, Elana Pinchefsky, Robyn Whitney, Diane Wilson, Steven P. Miller, Miguel A. Cortez

**Affiliations:** ^1^Department of Pediatrics, Divisions of Neonatology, McMaster Children's Hospital, McMaster University, Hamilton, ON, Canada; ^2^Child Health Evaluative Sciences Research Program, Toronto and Cancer Care Ontario, The Hospital for Sick Children, SickKids Research Institute, Toronto, ON, Canada; ^3^Department of Pediatrics, Divisions of Neurology, Hospital for Sick Children, University of Toronto, Toronto, ON, Canada; ^4^Department of Pediatrics, Temerty Faculty of Medicine, University of Toronto, Toronto, ON, Canada; ^5^Department of Pediatrics, Division of Neurology, Centre Hospitalier Universitaire Sainte-Justine, University of Montreal, Montreal, QC, Canada; ^6^Department of Pediatrics, Divisions of Neurology, McMaster Children's Hospital, McMaster University, Hamilton, ON, Canada; ^7^Department of Pediatrics, Divisions of Neonatology, Hospital for Sick Children, University of Toronto, Toronto, ON, Canada; ^8^Department of Pediatrics, Faculty of Medicine, University of British Columbia, Vancouver, BC, Canada

**Keywords:** infantile spasm (IS), heart rate variability, perinatal brain injury, autonomic dysfunction, hypsarrhythmia

## Abstract

**Background:**

Infantile epileptic spasm syndrome (IESS) is an age-dependent epileptic encephalopathy with a significant risk of developmental regression. This study investigates the association between heart rate variability (HRV) in infants at risk of IESS and the clinical onset of IESS.

**Methods:**

Sixty neonates at risk of IESS were prospectively followed from birth to 12 months with simultaneous electroencephalogram (EEG) and electrocardiogram recordings for 60 min at every 2-month interval. HRV metrics were calculated from 5 min time-epoch during sleep including frequency domain measures, Poincare analysis including cardiac vagal index (CVI) and cardiac sympathetic index (CSI), and detrended fluctuation analysis (DFA α1, DFA α2). To assess the effect of each HRV metric at the 2-month baseline on the time until the first occurrence of either hypsarrhythmia on EEG and/or clinical spasm, univariate cox-proportional hazard models were fitted for each HRV metric.

**Results:**

Infantile epileptic spasm syndrome was diagnosed in 20/60 (33%) of the cohort in a 12-month follow-up and 3 (5%) were lost to follow-up. The median age of developing hypsarrhythmia was 25 (7–53) weeks and clinical spasms at 24 (8–40) weeks. Three (5%) patients had clinical spasms without hypsarrhythmia, and 5 (8%) patients had hypsarrhythmia before clinical spasms at the initial presentation. The infants with high CSI (hazard ratio 2.5, 95% CI 1.2–5.2, *P* = 0.01) and high DFA α1 (hazard ratio 16, 95% CI 1.1–240, *P* = 0.04) at 2 months were more likely to develop hypsarrhythmia by the first year of age. There was a trend toward decreasing CSI and DFA α1 and increasing CVI in the first 8 months of age.

**Conclusion:**

Our data suggest that relative sympathetic predominance at an early age of 2 months may be a potential predictor for developing IESS. Hence, early HRV patterns may provide valuable prognostic information in children at risk of IESS allowing early detection and optimization of cognitive outcomes. Whether early intervention to restore sympathovagal balance *per se* would provide clinical benefit must be addressed by future studies.

## 1. Introduction

Infantile epileptic spasm syndrome (IESS) is an age-dependent epileptic syndrome manifested as clusters of clinical spasms along with a characteristic electroencephalogram (EEG) pattern. It affects 2.9/10,000 children within the first year of age (1). Irrespective of the variations in clinical presentation such as West syndrome, single-spasm variant, hypsarrhythmia without spasms, and spasms without hypsarrhythmia, diagnosis is often associated with developmental arrest/regression and profound cognitive impairment (1–3). Among the very few cases in which the triggering event can be adequately identified, the latent period between the triggering event and the onset of spasms usually ranges from 1 to 11 months (4). Therefore, prognostic biomarkers that may be indicative of disease onset in patients at risk of IESS during the latent period are needed.

Cardiac autonomic dysfunction has been reported in patients with epilepsy (5, 6). Specifically, high sympathetic tone ictally and interictally has been implicated as a cause of sudden unexpected death in epilepsy (SUDEP) (7, 8). Similarly, IESS has also been associated with autonomic dysregulation (9–13). Heart rate variability (HRV), a surrogate marker of autonomic functional state, was reduced in patients with IESS and predicts increased risk for subsequent cardiac events (13). Patients with IESS also showed the absence of circadian variability in heart rate (9). Patients in the early phase of the disease showed the presence of hypsarrhythmia coincided with reduced HRV measures in the awake state compared to controls (12). Moreover, treatment with adrenocorticotrophic hormone significantly increased cardiac vagal activity resulting in bradycardia within 7 days of initiation which then disappeared within a week of treatment cessation (10). These effects are believed to be mediated through the pro-opiomelanocortin-positive neurons of the arcuate nucleus of the hypothalamus and the nucleus of the tractus solitarius of the medulla (14). On the contrary, Jansen et al. suggested that the mere onset of epileptic encephalopathy is not sufficient to alter spectral components of heart rate but chronic autonomic changes appeared after 3 years of epilepsy (11). Although these findings indicate that autonomic alterations can be found in IESS, convincing evidence is not yet available.

Heart rate variability analysis quantifies the fluctuations in RR intervals (RRi) from electrocardiographic (ECG) signals. This variability is mediated by sympathetic and parasympathetic efferent activity in the heart and can provide information on the functional state of the autonomic nervous system (ANS) (15). It is believed to be an emergent property of interdependent regulatory systems operating on different time scales to maintain homeostasis (16). Understanding early physiological biomarkers that can be linked to disease onset and evolution is of great interest in IESS research. The goal of this study was to characterize the temporal changes in HRV in a cohort of infants at-risk of IESS. We compared the HRV before and after the onset of clinical features in infants symptomatic for IESS with infants who do not develop the disease. Furthermore, we investigated whether certain early HRV metrics may be predictive of the clinical onset of the disease or hypsarrhythmia in the first year of age. We hypothesized that infants who develop IESS will show an altered autonomic functional state prior to or at the onset of symptoms.

## 2. Methods

### 2.1. Study design

We studied a prospective cohort of infants with risk factors for IESS at The Hospital for Sick Children, Toronto, Canada. Diagnoses considered at risk are listed in Supplementary Table S1. Patients were enrolled between birth and a postmenstrual age of 2 months, at discharge from NICU, during emergency room visits, or Pediatric Neurology clinic visits. The first EEG was performed at the postmenstrual age of 2 months. Subsequently, the cohort was longitudinally followed by bi-monthly EEG recording until the postmenstrual age of 12 months. Institutional research ethics board approval was obtained, and informed consent was obtained from the parents of all subjects.

### 2.2. Patients

All eligible infants were enrolled in the study from June 2016 to March 2019. Infants who develop clinical spasms and/or showed hypsarrhythmia on the EEG were diagnosed with IESS. Infants who did not develop either event by 12 months were considered the control group. Patients were excluded if they had congenital heart disease or if they were prescribed antiarrhythmic agents due to effects on HRV or were lost to follow-up.

### 2.3. EEG analysis

All EEGs were of a minimum duration of 60 min using the international 10/20 system for electrode placement and were recorded on the NicoletOne™ vEEG system (Natus, Middleton, WI). All had simultaneous video recordings to document clinical events. Each EEG was analyzed independently by two Canadian certified clinical neurophysiologists (VC, MAC), with 98% reliability and available for final review, if any disagreement occurred, prior to the longitudinal EEG protocol with phase synchrony and variability analyses to detect the development of the early changes seen in “hypsarrhythmia” (17). Early abnormal EEG features were classified as pre-hypsarrhythmia (EEG type 1), modified hypsarrhythmia (EEG type 2), and hypsarrhythmia (EEG type 3) based on Philippi's classification, using the international 10–20 system of electrode placement, Pz reference, at 30 mm/s (18).

### 2.4. ECG processing

Electrocardiographic data were sampled at 256 Hz and a 5 min segment of ECG recordings from seizure-free epoch during NREM sleep was extracted. Signal processing was performed using the Kubios HRV analysis software, version 3.3. A built-in QRS detection algorithm based on the Pan–Tompkin's algorithm was applied to detect R-peaks and RRi was computed. Cubic spline interpolation at a rate of 4 Hz generated an equidistantly sampled RR time series. An automatic artifact correction algorithm was used to detect technical and physiological artifacts and remove ectopic/misplaced beats from the normal sinus rhythm. A detrending method based on smoothness prior to regularization with a cutoff frequency of <0.04 Hz was used to remove non-stationarities. Subsequently, fast-Fourier transformation separated the RR time series into its component frequencies, quantified as power spectral density (ms^2^), i.e., the area under the curve (AUC) in a given segment of the spectrum was estimated by Welch's periodogram method using a window width of 300 s with 50% overlap and a smoothing window of 0.02 Hz.

### 2.5. HRV analysis

**Figure 1:**
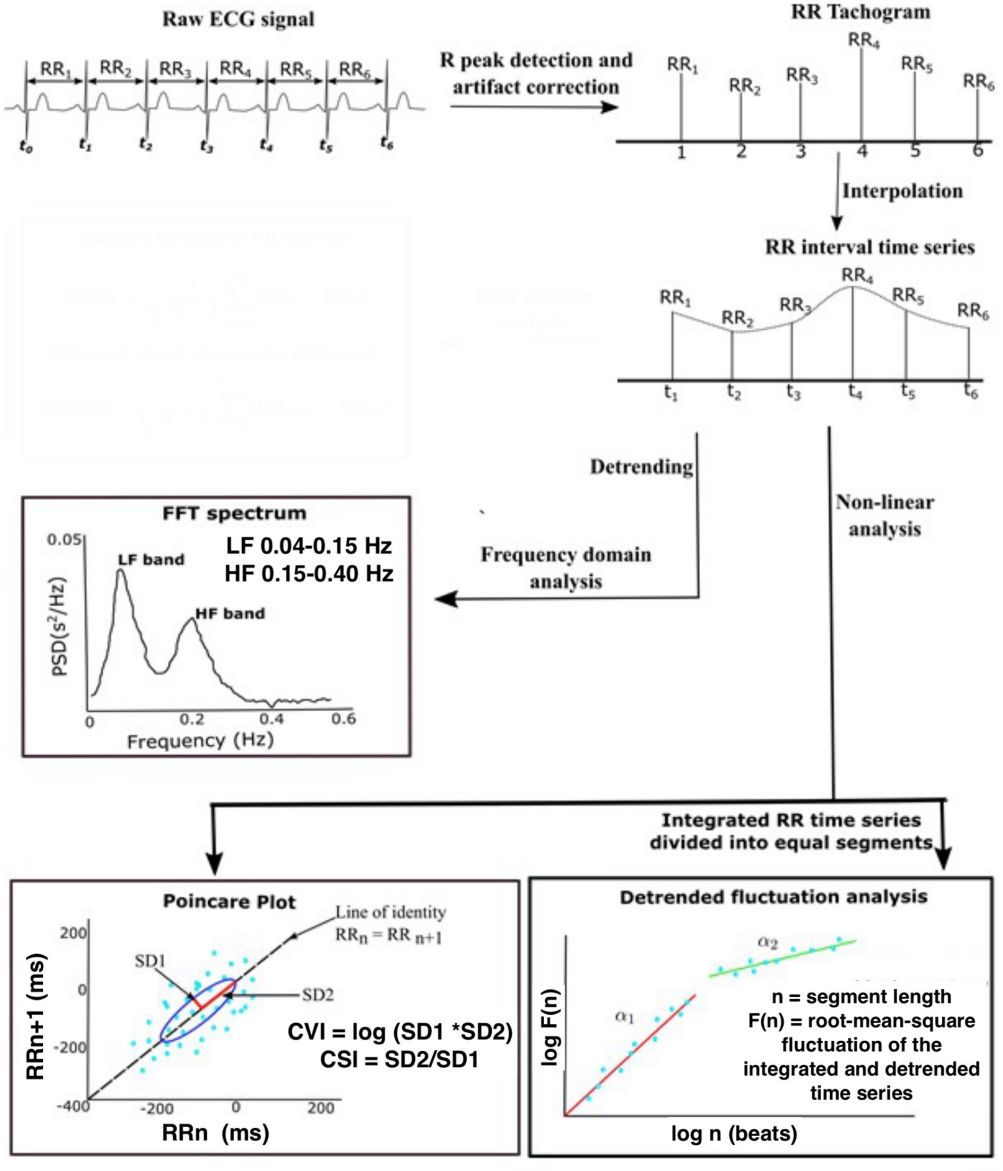
Schematic demonstrating the steps in the analysis of heart rate variability. Raw ECG data were sampled at 256 Hz and the 5 min segment of ECG recordings was extracted from seizure-free epochs during NREM sleep. Pan–Tompkin's algorithm was applied to compute RRi and generate an equidistantly sampled RR time series. Standard frequency domain HRV measures included normalized power of variability in two standard frequency bands HF (0.15–0.4 Hz) and LF (0.04–0.15 Hz). The Poincaré plot is a scatter plot representing the correlation between consecutive RRi. SD1 represents the standard deviations of the points perpendicular to the line of identity, while SD2 represents the standard deviation of the points along the line of identity. DFA measures the correlations within the data for different time scales. Variability is characterized by the slope of the regression line obtained as alpha1 (range of 4–16 beats) and alpha 2 (range of 16–64 beats). The slope ranges in value from 0.5 (random) to 1.5 (correlated). ECG, electroencephalogram; NREM, Non-Rapid eye movement sleep; R-R, R-R peak interval; FTT, Fast Fourier transform; HF, High-frequency filter; LF, Low-frequency filter; CVI, cardiac vagal index; CSI, cardiac sympathetic index; SD1, Standard deviation direction perpendicular; SD2, standard deviation direction diagonal. Adapted from Goswami et al. (23).

Standard frequency domain HRV measures included normalized power of variability in two standard frequency bands HF (0.15 to 0.4 Hz) and LF (0.04–0.15 Hz) (19). In this study, we used a Poincaré plot and Detrended fluctuation analysis (DFA). The Poincaré plot is a scatter plot representing the correlation between consecutive RRi. Poincaré analysis quantifies the dispersion in the RRi in the direction perpendicular (SD1) and diagonal (SD2) to the line of the identity of the scatter plot, respectively (15, 20). SD1 represents the standard deviations of the points perpendicular to the line of identity (a measure of short-term variability), while SD2 represents the standard deviation of the points along the line of identity (a measure of overall variability) (21). Cardiac vagal index (CVI = log_10_ SD1 X SD2) and cardiac sympathetic index (CSI = SD2/SD1) are further derived measures that assess parasympathetic and sympathetic function independently and simultaneously (22). DFA measures the correlations within the data for different time scales. The RRi time series is integrated prior to fragmentation into segments of equal length and a least squares line is fitted into each segment. Next, the integrated series is detrended by computing the root-mean-square fluctuation of the local trend within each segment from the integrated time series. This process repeated over different segment lengths yields a log graph of fluctuation as a function of segment length (Figure 1). Variability is characterized by the slope of the regression line obtained as alpha1 (range of 4–16 beats) and alpha 2 (range of 16–64 beats). The slope ranges in value from 0.5 (random) to 1.5 (correlated).

### 2.6. Statistical analysis

Demographic information was summarized using means and standard deviations or medians and IQR where appropriate. Comparisons of data between the patients who were diagnosed with IESS and those who did not experience the event (the group without IESS) were made using the independent Student *t*-test for continuous variables and the chi-square test for categorical variables. To assess the effect of each HRV metric measured at baseline (2 months) on the time until the first occurrence of an event (either hypsarrhythmia on EEG or clinical spasm), univariate cox-proportional hazard models were fitted for each HRV metric. Analysis was performed using R version 4.0.3 (10 October 2020).

## 3. Results

Sixty infants (29 girls and 31 boys) were enrolled in the study. IESS was diagnosed in 20/60 (33%) of the cohort at a 12-month follow-up. Three (5%) infants were lost to follow-up. Only 17/20 infants with a diagnosis of IESS had ECG data available at 2 months (baseline). There was no significant difference between the perinatal characteristics of infants who received and did not receive a diagnosis of IESS except the prevalence of hypoxic-ischemic encephalopathy was less frequent in the group that developed IESS (Table 1). The clinical characteristics of the 20 patients with the diagnosis of IESS are shown in Table 2. The median age at the onset of hypsarrhythmia was 25 (7–53) weeks and the onset of clinical spasms was 24 (8–40) weeks. Three patients had clinical spasms without hypsarrhythmia, showing only mildly slow background activity, and five patients had hypsarrhythmia before the onset of clinical spasms. Among 12 patients who presented with both clinical spasms and hypsarrhythmia, six patients presented with a Type 2 EEG pattern at the initial diagnosis, out of which two patients later progressed to a Type 3 EEG pattern. Five patients presented with a Type 3 EEG pattern at the initial diagnosis.

**Table 1:** Clinical characteristics of the study population.

		**Diagnosed with infantile spasm by 12 months (*n =* 20)**	**Not diagnosed with infantile spasm by 12 months (*n =* 37)**	***p* value**
Gestational age at birth (weeks)^*^		39.5 ± 1.4	38.7 ± 3.9	0.15
Male gender, n (%)		7 (41)	21 (52)	0.26
Preterm birth, n (%)		0 (0)	6 (15)	0.89
Maternal age (years)*		30.0 ± 4.4	31.5 ± 5.3	0.56
Birth weight (g)^*^		3245 ± 509	3292 ± 998	0.68
Hypoxic ischemic encephalopathy, n (%)		1 (7)	16 (40)	0.02
Metabolic disorders, n (%)		3 (17)	4 (10)	0.18
Hypoglycemic brain injury, n (%)		5 (30)	13 (32)	0.09
Ethnicity, n (%)	African/American	0 (0)	5 (12)	0.30
	Asian	9 (53)	7 (17)	
	Caucasian	5 (29)	15 (38)	
	Others	3 (17)	13 (32)	

^*^Mean (standard deviation).

**Table 2:** Patient profiles of cases with infantile spasms.

**Patient no**.	**Primary etiology**	**Additional diagnosis**	**Age of onset of spasm**	**Age of onset of hypsarrythmia**	**Medication**
1	HIE	Global developmental delay	6 months	5 months	LEV, VIG
2	HIE	Microcephaly, neonatal seizures, Global developmental delay	6 months	10 months	PHB, LEV, TOP
3	SCN2A	Microcephaly, epileptic encephalopathy, Global developmental delay, cortical visual impairment		4 months	KD, LEV, PHE, PYR
4	Nonketotic Hyperglycinemia	Global developmental delay	2 months	4 months	LEV, TOP, PHB
5	Aicardi syndrome	Congential Zika virus infection, Global developmental delay, ventriculomegaly	8 months		LEV
6	Neonatal hypoglycemia	Cystic encephalomalacia, neonatal seizures, Global developmental delay	7 months	8 months	PHB, VIG
7	Molybdenum co-factor deficiency	Cystic encephalomalacia, refractory seizures		10 months	PHB, CAR, CLOB, LEV, GBP
8	Extensive bilateral polymicrogyria	Microcephaly	12 months	12 months	VIG
9	Holoprosencephaly	Chromosome 18p deletion syndrome, Laryngomalacia, Cleft palate	4 months	4 months	VIG, TOP
10	Trisomy 21	Down Syndrome	4 months	4 months	VIG
11	Tuberous Sclerosis	Rhabdomyoma	4 months	10 months	VIG
12	Neonatal hypoglycemic brain injury	Status epilepticus, Laryngomalacia, Hypertension	6 months	6 months	PHB, VIG
13	Neonatal hypoglycemic brain injury	Seizures in newborn, feeding difficulty	6 months	6 months	LEV, PHB, VIG, PR, TOP
14	Aicardi syndrome	Pierre Robin syndrome	3 months		VIG, LEV
15	Neonatal Herpes simplex	Neonatal seizure	6 months	6 months	VIG
16	Aicardi syndrome	Micropthalmia	2 months	2 months	PHB, LEV, VIG, PR
17	Ohtahara Syndrome	Laryngomalacia		2 months	PHB, LEV, TOP, VIG, CAR
18	Ohtahara Syndrome			4 months	PB, ClOB, VGB, PHE
19	Microcephaly	Anemia	3 months		PB, VGB
20	Neonatal hypoglycemic brain injury		6 months	6 months	LEV, PB, VGB, PR, TOP

HIE, Hypoxic Ischemic Encephalopathy; LEV, Levitiracetam; PHB, Phenobarbitone; VIG, Vigabantrin; PR, Prednisolone; CAR, Carbamazepine; PHE, Phenytoin; TOP, Topiramate; KD, Ketogenic diet; PYR, Pyridoxine; CLOB, Clobazam; GBP, Gabapentin.

**Figure 2:**
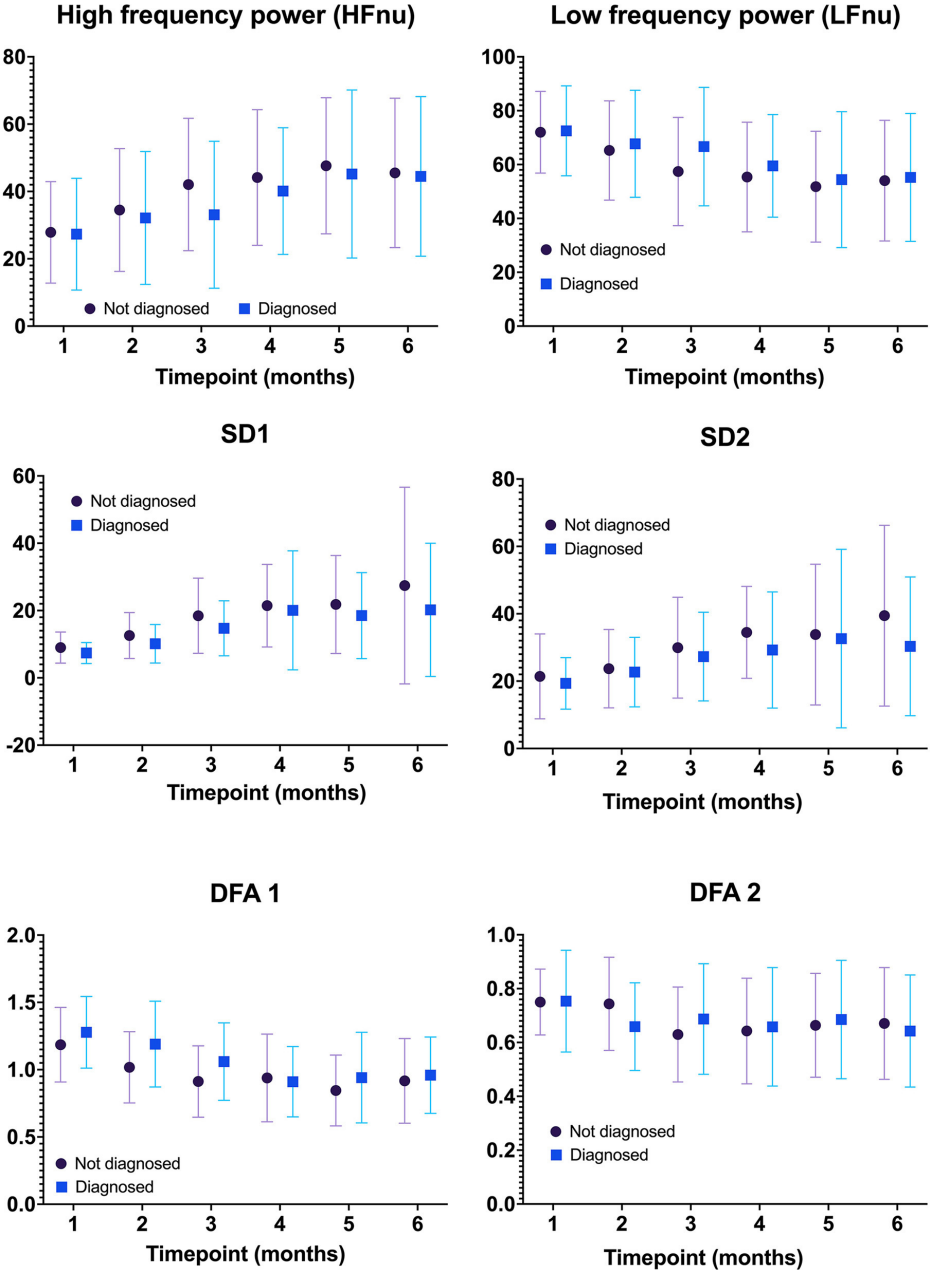
Comparison of HRV metrics between infants with and without the diagnosis of IESS at each age group. There was no statistically significant difference in any HRV metric between infants with and without the diagnosis of IESS at all time points tested. IESS, Infantile epileptic spasm syndrome; HRV, heart rate variability.

**Figure 3:**
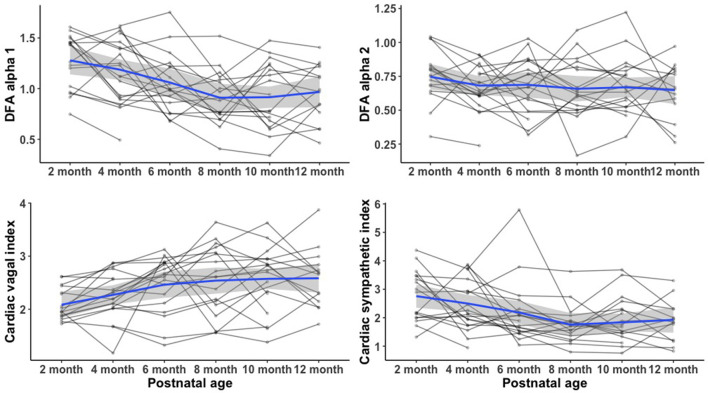
Heart rate variability trajectory at 1 year of age in infants with IESS. There was a trend toward an increase in mean CVI values at consecutive visits until the postnatal age of 8 months after which there was a plateau. There was also a simultaneous decrease in CSI and DFA α1, which also plateaus around 8 months. IESS, Infantile epileptic spasm syndrome; HRV, heart rate variability; CVI, cardiac vagal index; CSI, cardiac sympathetic index; DFA α1, detrended fluctuation analysis alpha 1.

Figure 2 compares the mean (SD) of the frequency domain measures (HF and LF), Poincaré analysis measures (SD1 and SD2), and detrended fluctuation analysis measures (DFA1 and DFA2) of HRV metrics between infants with and without the diagnosis of IESS at each age group. At each time point in the first year of age, the mean difference in all HRV variables between the two groups was not statistically significant. Figure 3 shows the individual trajectories of HRV variables in infants with the diagnosis of IESS during the first year of age. Overall, there was a trend toward an increase in mean CVI values at consecutive visits until the postnatal age of 8 months followed by a plateau. There is also a simultaneous decrease in CSI and DFA α1, which also plateaus around 8 months. When the mean CVI and CSI values on the ECG recording in the visit preceding the visit that showed evidence of hypsarrhythmia or clinical spasm (whichever was earlier) were compared with the ECG recording taken at the visit with the diagnosis, the difference was not statistically significant (Supplementary Figure S1).

The predicted probability of an event, i.e., diagnosis of clinical spasm or hypsarrhythmia in the first year of age, at the first visit (2 months) for each patient was obtained using a univariate Cox proportional hazard model, and for each biomarker, the hazard ratio corresponding to a one unit increase in the biomarker value and its 95% confidence interval are presented in Table 3. Only CSI (hazard ratio 2.5, *P* = 0.01) and DFA α1 (hazard ratio 16, *P* = 0.04) measured at baseline reached a statistical significance, indicating that increasing values of these markers at 2 months are associated with increased risk of having a diagnosis of IESS by 1 year of age. We tested the classification model using the receiver operating characteristic curve, which showed the AUC for CSI [AUC 0.61 (95% CI 0.43–0.78)] and DFA alpha 1 [AUC 0.60 (95% CI 0.43–0.79)].

**Table 3:** Results of univariate cox-proportional regression model.

**HRV metric**	**Beta coefficient**	**Hazard ratio (HR)**	**95% Confidence interval**	***p* value**
**Time to diagnosis of infantile epileptic spasm syndrome (IESS)**				
CVI	−0.23	0.8	0.2–3	0.76
CSI	0.92	2.5	1.2–5	0.01
DFA alpha 1	2.8	16	1.1–240	0.04
DFA alpha 2	0.36	1.4	0.02–110	0.87

## 4. Discussion

We examined the evolution of HRV parameters in patients at risk of developing IESS during the first year of age and observed no statistically significant difference in any HRV metric between infants who are diagnosed with IESS and who are not in any age group. However, infants who had the diagnosis of IESS at any point before 12 months of age tend to have higher CSI and DFA α1 at a 2-month visit. These findings suggest that non-linear and complex measures of HRV may provide added value in the interpretation of the early heart rate patterns in infants with perinatal risk factors for IESS.

Heart rate variability analysis has been widely used as a non-invasive surrogate of cardiac autonomic balance in children with a variety of pathological conditions, including congenital heart disease, attention deficit hyperactivity disorder, and type I diabetes (24–26). Autonomic dysfunction, especially reduced parasympathetic activity, has previously been reported in patients with different types of epileptic syndromes. The commonly reported HRV parameters include lower LF and HF in refractory epilepsy (27), lower SDNN in the interictal phase in patients with partial seizures and generalized seizures (8, 28, 29), and lower LF/HF ratio in patients with complex partial seizures compared to controls (7, 30). Similarly, parasympathetic withdrawal has also been reported in children (aged 0–2 years) with hypertonia with disruption of normal physiological increase in parasympathetic HR modulation during early life (31). IESS is a unique electroclinical syndrome on its own and, recently, there has been an interest in understanding the autonomic regulation in infants with IESS especially before and after treatment.

Few studies have assessed altered cardiac autonomic function in patients with IESS using HRV. Moller et al. (12) reported from a cohort of 23 patients that in the initial phase, the presence of hypsarrhythmia is associated with reduced measures of SDNN and total power in an awake state compared to healthy controls. Hattori et al. (10) also reported from a cohort of 15 patients that the diagnosis was associated with significantly higher LF power compared to controls (10). Interestingly, the distribution of LF power in the patients and controls overlapped, indicating that not all the patients with West syndrome had autonomic changes (10). The effect of IESS on LF power and LF/HF ratio is rather controversial, with studies showing contradictory results (10, 12). Using longer ECG time epochs (24 h), Gencpinar et al. reported both reduced sympathetic and parasympathetic activity in patients with IESS (13). Ardura et al. (9) reported a patient with IESS who did not have any circadian variability in heart rate. Overall, HRV studies using both short (5 min) and long (24 h) epochs have demonstrated alterations in different time and frequency domain measures in infants with infantile spasms. Contrary to their findings, we did not observe any statistically significant difference in the frequency domain HRV measures between the infants with and without the diagnosis at any age group. This might be because the control group in our study was not a healthy cohort unlike other studies and the entire cohort had some perinatal risk factors for developing epilepsy. Because autonomic dysfunction may occur at the beginning of the clinical presentation (12), we also compared the HRV measures at the visit at which the hypsarrhythmia (Type 2 or 3 patterns) was present in EEG or clinical spasm was reported with the HRV measures of the preceding visit. The mean difference in all HRV measures did not reach statistical significance, suggesting that reduced HRV associated with IESS may not be present before the onset of symptoms. Also, since our cohort was prospectively followed and treatment started promptly upon diagnosis, the autonomic dysfunction documented in prior studies, which recruited patients after the onset of clinical symptoms, was not evident. Similarly, such abnormal autonomic cardiac influence is transient, related to epileptiform activity, and is reversible with the initiation of treatment (10, 12).

The interaction between seizures and the ANS is complex. The insula, anterior cingulate gyrus, and ventromedial prefrontal cortex all affect cardiac rate, rhythm, and output, respectively (32). Patients with epilepsy experience long-lasting changes in autonomic regulation and its target organs, especially the heart. In addition, autonomic dysregulation due to epilepsy is suspected to contribute to SUDEP (33). Compared to traditionally reported time and frequency domain HRV indices, there is now emerging evidence around complexity analysis of HRV as more precise predictors of adverse outcomes in a variety of patient groups (34, 35). For example, significant changes in DFA values were demonstrated in the absence of altered time and frequency domain measures before the onset of paroxysmal atrial fibrillation in patients without structural heart disease (35). Interestingly, our data indicating CSI and DFA α1 at 2 months are the only predictors of the risk of developing IESS by 1 year among all tested HRV-based biomarkers.

The significance of high values of CSI and DFA α1 in early infancy may be explained with referral to pre-clinical studies. According to Toichi et al. (22), the CSI reflects cardiac sympathetic activity unaffected by vagal activity. On the contrary, CVI is a sensitive index of cardiac vagal function that is not affected by sympathetic activity. Therefore, our findings indicate that among the infants with perinatal risk factors for epilepsy, the infants who are noted to have sympathetic overactivity at 2 months are most likely to develop IESS by 1 year. The DFA analysis method is based on the fractal theory used to determine the statistical self-affinity along the time and value axes of a biological signal (36). Fractal objects are characterized by a self-similar structure in which each part has characteristics resembling the whole. The self-similar (fractal) nature of HRV manifests in strong autocorrelations within the signal, which decay slowly with time. The range of these autocorrelations is indicated by DFA α1, the slope of the log detrended fluctuation vs. log window size (4–16 beats). HRV is determined by two discrete regulatory systems: (i) ANS and its interaction with (ii) the sinoatrial node. The sinoatrial node has an intrinsic intercellular mechanism (“coupled-clock” system) that oscillates even without neural input (37). Using DFA analysis, the autonomic signature for relative contributions of the individual systems (ANS and sinoatrial node) has been delineated (37). The sinoatrial node shifts the signal dynamics from pink noise (DFA α1 = 1) to Brownian noise (DFA α1 = 1.5), indicating that most of the complexity is found at the longest time scales and the long-range variability in the beat intervals. On the contrary, ANS contribution to the beat-interval time series is modeled as a stochastic process with white noise characteristics (DFA α1 = 0.5), and periodic components at frequencies corresponding to the respiratory sinus arrhythmia and baroreceptor reflex influencing short-term variability (37). Therefore, taken together, the group of infants who show the early HRV pattern and develop IESS exhibit characteristic long-range patterns with decreased short-term variability [sinoatrial node predominance] at 2 months compared to the group of infants who do not.

Beyond that, we found a trend toward decreasing CSI and DFAα1 over time until 8 months with a simultaneous increase in CVI measures. We speculate that these transient alterations of physiologically relevant regulatory mechanisms toward relative sympathetic predominance may be mechanistically similar to dysautonomia noted after a variety of acute cerebral insults, termed “paroxysmal sympathetic hyperactivity” (38). Sympathetic overactivity increases intracellular cyclic AMP and raises the rate of action potential generation in the sinoatrial node, further supporting our interpretation of sinoatrial node predominance in heart rate regulation. The fractal organization of heart rate dynamics is determined by a delicate interplay between sympathetic and vagal outflow, with more random dynamics occurring during the coactivation of sympathetic and vagal outflow (39). Previously thought to be epileptogenic in nature (38), current hypotheses of paroxysmal sympathetic hyperactivity revolve around “disconnection theory”, namely, (i) brainstem excitatory centers are released from higher control with a resultant hyper-sympathetic state or alternatively, (ii) an excitatory/inhibitory ratio model suggests that causative brainstem centers are inhibitory in nature and that sympathetic hyperactivity originates at the spinal cord level in a process analogous to autonomic dysreflexia following high thoracic spinal cord injury (40). Since the most common precedents of IESS were hypoxic-ischemic encephalopathy, chromosomal anomalies, malformation, perinatal stroke, hypoglycaemic brain injury, tuberous sclerosis, periventricular leukomalacia, genetic mutations along GABAergic forebrain dorsal–ventral development, and synaptic pathways (1, 41), it is likely that alterations of the fractal heart rate dynamics are preceded by unbalanced neuro-autonomic inputs. With age, a corresponding increase in CVI also indicates an increase in short-term variability and vagal influence on the HRV structure. Present results confirm the findings reported by Schechtman et al. (42) that cardiac vagal modulation increases with age in healthy subjects.

Our study is limited by sample size and the results might potentially be underpowered due to the small sample size. However, given the low prevalence of the disease in question, it is comparable with other studies in the field. We selected patients with perinatal risk factors for epilepsy that limited the generalization of the result to patients with either congenital malformations, genetic abnormalities, or perinatal brain injury. Thus, healthy infants, in whom the fluctuation of heart rate would be different, were not part of the control group. However, the controls have an overrepresentation of hypoxic-ischemic damage, and some of them were premature which may contribute to relative sympathetic dominance at 2 months of age. The differences noted in sympathovagal balance may be affected by the differences in clinical diagnosis, especially the presence of syndromes related to the IESS group (e.g., SCN2A, tuberous sclerosis, Ohtahara syndrome, and polymicrogyria) compared to the control group and/or different maturity of the ANS and sleep patterns of 2 months old. The limitations of sample size that preclude subgroup and multivariate analyses to investigate confounders and etiological effects can be mitigated by a multicentred approach. Prospective longitudinal studies of IESS require larger catch ascertainments to acquire the sample sizes necessary to make comprehensive conclusions due to the rarity of IESS in the population (3). The time epochs selected for our analysis were 5 min; thus, it may not capture long-term variability and time domain measures efficiently. Previous studies have validated the use of 5–10 min for non-linear and frequency domains of HRV analysis. This study demonstrates the value of complexity measures of HRV in evaluating the autonomic function, especially given the concerns raised around the difficulty in interpretation of relative contributions of parasympathetic and sympathetic arms of ANS to HRV. Our prospective approach also raises the hypothesis that autonomic dysfunction may be transient and possibly preventable if diagnosed early with the timely intervention of therapy. The early dysautonomia that we observed in infants who are most likely to be symptomatic in the first year of age improves with age, implying that HRV measures considered in future studies should take into account the age-related changes in heart rate dynamics with postnatal age. In addition, selective HRV measures may be potentially a tool for risk stratification and patient selection for closer follow-up. Avoiding or minimizing the duration of untreated hypsarrhythmia may decrease the burden of the disease and optimize the neurodevelopmental outcomes of children at risk. The early predictors of IESS identified in the current study are identified based on a small sample size which limited the use of multivariate analysis to adjust for potential confounders. Therefore, the results found in this study would need validation in larger samples.

## 5. Conclusion

In the present study, we introduced the possibility of using complexity measures of heart rate variability to evaluate the early cardiac autonomic function, which may provide a window to detect infants more susceptible to developing IESS among children at risk of the disease. Increased DFAα1 and CSI at 2 months are potential early predictors for higher risks of epileptic syndrome. Whether early intervention to restore sympathovagal balance *per se* would provide clinical benefit must be addressed by future studies.

## Data availability statement

The raw data supporting the conclusions of this article will be made available by the authors, without undue reservation.

## Ethics statement

The studies involving human participants were reviewed and approved by Research Ethics Board-SickKids. Written informed consent to participate in this study was provided by the participants' legal guardian/next of kin.

## Author contributions

IG: HRV analysis and manuscript preparation. BM: statistical analysis. VC and MC: EEG analysis and critical review. ET: critical review of the manuscript. EP, RW, and DW: data collection. SM: critical review of the manuscript. MC: conceptualization. All authors contributed to the article and approved the submitted version.
