# Supplementary material for: Early transient dysautonomia predicts the risk of infantile epileptic spasm syndrome onset: A prospective cohort study
Source: Front Neurol. 2022 Dec 22;13:1090155. doi: 10.3389/fneur.2022.1090155 (PMC9815183; doi:10.3389/fneur.2022.1090155)
Supplement: Supplementary file 1 [file Table_1.DOCX]

**Table SI: List of conditions considered to be a risk factor for Infantile spasms**

All male and female neonates (from 0 month to 1 month) or infants (from 1 month and 1 day to 2 months) with identified risk factors including the following:

| Prenatal malformations or cryptogenic | Chromosomal or Genetic abnormality | Transient metabolic | Vascular | Metabolic or endocrine |
| --- | --- | --- | --- | --- |
| - Agenesis of the corpus callosum - Agyria/polygyria - Cortical dysplasia - Schizencephaly - Heterotopia - Holoprosencephaly - Lissencephaly - Hydrocephalus - Microcephaly - Dandy Walker malformation - Optic nerve hypoplasia - Incontinentia pigmenti - Neurofibromatosis - Tuberous sclerosis complex - Hypomelanosis of Ito - Arachnoid cyst | - Down syndrome - XXY - 22q deletion - 17p 13.3 microdeletion - 1p36 del - del 1q36 1ptel - Muscle eye brain disease. - Genetic mutations: ARX,CDKL5,FOXG1,GRIN1/2A,MAGI2,MEF2C,SLC25A22,SPTAN1,STXBP1 | - Hypoglycemic brain jury - Hypoxic Ischemic Encephalopathy (HIE) | - Stroke - Periventricular leukomalacia of prematurity - Moyamoya disease | - Classical Phenylketonuria - Organic acidurias - Amino acidurias - Mitochondrial disorders - Pyridoxine deficiency - GAMT and AGAT deficiencies |
