# Supplementary figures and images for: Early transient dysautonomia predicts the risk of infantile epileptic spasm syndrome onset: A prospective cohort study
Source: Front Neurol. 2022 Dec 22;13:1090155. doi: 10.3389/fneur.2022.1090155 (PMC9815183; doi:10.3389/fneur.2022.1090155)

**Figure S2: Comparison of HRV at the diagnostic EEG-ECG with the recording at the preceding visit**


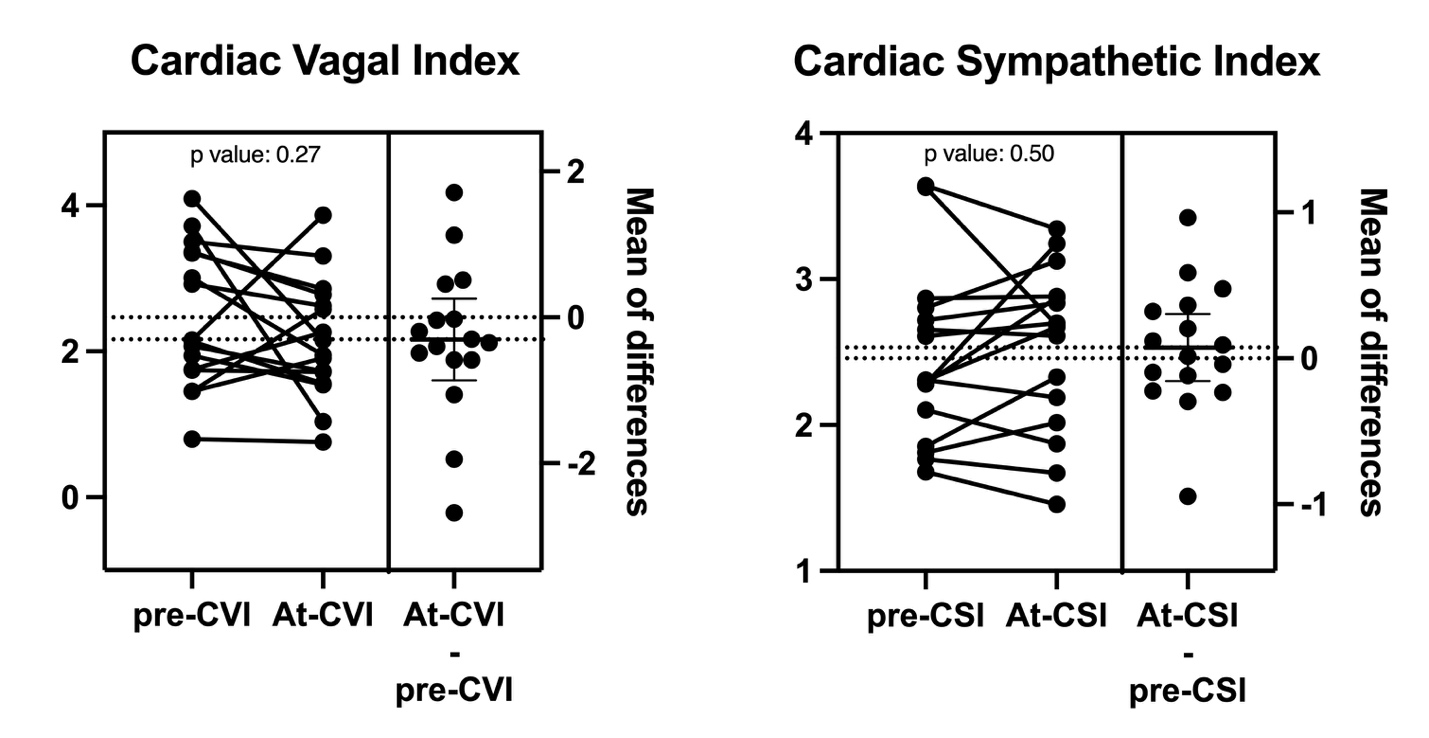

Supplement: Supplementary file 2 [file Table_2.DOCX]
